# Preparing for the next respiratory pathogen pandemic: using tabletop simulation exercises to strengthen national planning in Cook Islands, Costa Rica, Lebanon and Mongolia

**DOI:** 10.3389/fpubh.2024.1392894

**Published:** 2024-07-19

**Authors:** Hitesh Chugh, Oluwatosin Wuraola Akande, Roberto Arroba Tijerino, Moubadda Assi, Metua Bates, Atika Berry, Hebleen Brenes, Dulamragchaa Buyanbaatar, Urtnasan Chuluunbat, Gerelmaa Danzan, Oyungerel Darmaa, Ingrid Garcia, Nada Ghosn, Ruba Hikmat, Ana Maria Jimenez, Shakila Naidu, Karen Ngamata, Phuong Nam Nguyen, Beverley Paterson, Nomin-Erdene Tsogtgerel, Andrea Patricia Villalobos, Valentino Wichman, Kelly Safreed-Harmon, Shoshanna Goldin, Sylvie Briand, Gina Samaan

**Affiliations:** ^1^Epidemic and Pandemic Preparedness and Prevention, World Health Organization, Geneva, Switzerland; ^2^Epidemiology Unit, Health Surveillance Directorate, Ministry of Health, San José, Costa Rica; ^3^Pandemic Influenza Preparedness, World Health Organization, Beirut, Lebanon; ^4^Te Marae Ora, Ministry of Health, Avarua, Cook Islands; ^5^Preventive Medicine Department, Ministry of Public Health, Beirut, Lebanon; ^6^Institute for Research on Nutrition and Health, Ministry of Health, San José, Costa Rica; ^7^Health Emergency, World Health Organization, Ulaanbaatar, Mongolia; ^8^The National Influenza Centre, National Center for Communicable Diseases, Ministry of Health, Ulaanbaatar, Mongolia; ^9^Disease Prevention and Control and Risk Factors, Pan American Health Organization, Bogota, Colombia; ^10^Epidemiological Surveillance Unit, Ministry of Public Health, Beirut, Lebanon; ^11^Infectious Hazard Preparedness, World Health Organization, Cairo, Egypt; ^12^Communicable Diseases, Pan American Health Organization, San José, Costa Rica; ^13^Pacific Health Security and Communicable Diseases, World Health Organization, Suva, Fiji; ^14^Infectious Hazard Management, World Health Organization Regional Office for the Western Pacific, Manila, Philippines; ^15^National Centre for Epidemiology and Public Health, Australian National University, Canberra, ACT, Australia; ^16^Infectious Hazards Management Unit, Health Emergencies Department, Pan American Health Organization, Washington, DC, United States; ^17^Central Policy and Planning Office, Office of the Prime Minister, Avarua, Cook Islands; ^18^Independent Consultant, Barcelona, Spain

**Keywords:** pandemic, preparedness, response, exercise, respiratory, pathogen, planning

## Abstract

The Preparedness and Resilience for Emerging Threats (PRET) initiative takes an innovative mode-of-transmission approach to pandemic planning by advocating for integrated preparedness and response systems and capacities for groups of pathogens with common transmission pathways. The World Health Organization (WHO) launched this initiative in 2023 with the publication of PRET Module 1 addressing respiratory pathogens. Exercise PanPRET-1 is a customizable tabletop simulation exercise (TTX) package developed to complement PRET Module 1. The exercise scenario focuses on strengthening capacities for multisectoral coordination, risk communication and community engagement, and the triggers for operational decision-making. This article reports on the experiences of the first four countries to implement Exercise PanPRET-1: Cook Islands, Costa Rica, Lebanon and Mongolia. Exercise outcomes demonstrated that PanPRET-1 can be an effective tool for testing pandemic plans in a multisectoral forum and identifying opportunities to improve preparedness and response in key domains. In quantitative evaluations in Cook Islands, Costa Rica and Mongolia, high proportions of exercise participants indicated that multiple aspects of the exercise were well-designed and were beneficial for improving health emergency preparedness. Exercise participants in Lebanon provided qualitative feedback indicating that they found the exercise to be beneficial. Conducting a TTX and monitoring the implementation of action plans based on exercise findings facilitates a country-owned whole-of-society vision for pandemic planning. Countries are encouraged to incorporate TTX such as Exercise PanPRET-1 into a continuous cycle of activity to improve pandemic preparedness.

## 1 Introduction

The SARS-CoV-19 pandemic demonstrated the need for stronger preparedness and response systems to address a wide range of pathogens that may cause health emergencies (1). In early 2023, the World Health Organization (WHO) launched the Preparedness and Resilience for Emerging Threats (PRET) initiative, which takes an innovative mode-of-transmission approach to pandemic planning. In response to requests from WHO Member States (hereafter referred to as “countries”), PRET sets forth a whole-of-society vision for developing integrated preparedness and response capacities in relation to groups of pathogens that have common transmission pathways. The PRET initiative’s first module addresses respiratory pathogens including influenza viruses and coronaviruses (1). Since preparing for different types of respiratory pathogens requires similar actions, the mode-of-transmission approach leverages existing systems and capacities to maximize efficiency and coherence (1). Countries are encouraged to flexibly adapt guidance in PRET Module 1 to focus on the range of respiratory pathogens that are viewed as concerns in their respective contexts (1).

The PRET initiative emphasizes the importance of approaching pandemic preparedness in an iterative manner and engaging different sectors in updating and testing national respiratory pathogen pandemic plans (2). WHO has developed tools to facilitate the uptake of guidance in PRET Module 1, including a tabletop simulation exercise (TTX) package hereafter referred to as Exercise PanPRET-1. Simulation exercises are widely recognized as a useful tool for increasing government and societal readiness to respond to public health emergencies including disease outbreaks (3–5). A TTX brings together key stakeholders in a discussion-based setting to respond to a scripted emergency scenario and collectively address hypothetical events that realistically embody challenges associated with emergencies. According to the WHO Simulation Exercise Manual, a TTX “is designed to elicit constructive discussion between participants; to identify and resolve problems; and to refine existing operational plans” (5).

A TTX has multiple stages at which “injects” are presented to exercise participants asking them to address new developments associated with the emergency. Learnings that arise from the TTX are intended to guide measures to strengthen emergency preparedness. Governments and other actors have conducted TTX focusing on a wide range of pathogens such as coronaviruses (6), salmonella (7), Ebola virus disease (8), cholera (9), smallpox (10), and influenza viruses (11).

This article describes the implementation of Exercise PanPRET-1 in Cook Islands, Costa Rica, Lebanon and Mongolia. It reports on respective country outcomes and considers broader implications of these findings for national governments and other stakeholders. It also reflects on how countries can optimally integrate TTX activities into ongoing respiratory pathogen preparedness and response planning.

## 2 Developing and implementing the exercise package

### 2.1 Exercise package development

To help countries learn from pandemic and outbreak experiences, including from COVID-19, WHO staff developed Exercise PanPRET-1 drawing on information and templates in the *WHO Simulation Exercise Manual* (5). The overall objectives of the exercise package are “to deploy an adaptable tabletop simulation exercise package for countries to test/validate existing preparedness plans; update or develop a new plan; and/or advocate for respiratory pathogen preparedness planning.” The exercise package includes instructions for use, an adaptable draft scenario, templates for slide presentations, guides for facilitators and rapporteurs, and a participant evaluation form. WHO staff developed the package taking into account a range of country contexts, and incorporating best practices from related simulation exercises.

### 2.2 Exercise focus and content

Exercise PanPRET-1 focuses on three technical areas of respiratory pathogen preparedness and response: multisectoral coordination; risk communication and community engagement (RCCE); and triggers for operational decision-making.

The scenario presented in Exercise PanPRET-1 begins with an outbreak of a novel influenza virus among humans in a country on a different continent. The evolution of the situation is addressed in four stages, with each stage describing a series of events requiring government and societal action. For example, the first in-country cluster of cases of the disease is detected in stage two, misinformation is spreading in stage three, and hospitals are overwhelmed in stage four. Injects are presented at each stage, with accompanying questions asking exercise participants how their country’s preparedness and response systems would function as the scenario evolves.

### 2.3 Implementing the exercise

Exercise PanPret-1 was implemented in each country by an individual planning group. An exercise controller was appointed to coordinate the overall planning and delivery of the exercise with the assistance of the planning group. The exercise controllers for the Cook Islands, Costa Rica, and Mongolia were either WHO staff or WHO consultants. In Lebanon, the exercise controller was an external consultant recruited specifically for the exercise. Members of the planning group included representatives of the lead Ministry in charge of conducting the exercise (typically the Ministry of Health), the WHO Country Office, the WHO Regional Office, and technical staff from WHO Headquarters.

The planning group began the process by consulting with key stakeholders to determine the specific purpose, technical scope and desired outcomes of the exercise in their country context. The exercise controller and the planning group decided how to adapt the Exercise PanPRET-1 scenario to reflect their needs, taking into account each country’s existing health emergency management policy architecture.

The planning group invited representatives of different sectors to participate in the exercise, and sought to enlist participants from the governmental, private and civil society sectors to advance whole-of-society pandemic planning. Prior to implementation of the exercise, individuals who agreed to participate were provided background information including exercise objectives and current national pandemic plans.

Each country’s exercise was delivered by the exercise controller with assistance from facilitators and rapporteurs. The exercise concluded with a debriefing session whereby exercise and planning participants synthesized findings and identified lessons learned and recommendations. Following the exercise, lessons learned and recommendations were presented to government leaders, civil society and international partner agencies in order to galvanize actions to implement the recommendations.

This article reports on the experiences of the first four countries to implement Exercise PanPRET-1: Cook Islands, Costa Rica, Lebanon and Mongolia. Staff from WHO Country Offices, Regional Offices and Headquarters provided technical support to all four countries. Exercise participants in Cook Islands, Costa Rica and Mongolia completed an exercise evaluation survey. Exercise participants in Lebanon provided open-ended qualitative feedback on the exercise. Findings and lessons from each country (Table 1) were summarized using post-exercise reports that were shared with the exercise planning group and exercise participants.

**Table 1:** Characteristics of table-top simulation exercises in the four countries.

	Cook Islands	Costa Rica	Lebanon	Mongolia
Objective(s) of simulation exercise	Consider ways in which the national plan should be updated, based on (1) lessons from COVID-19, (2) updated WHO guidance, and (3) cross-cutting issues such as culture, language, gender, disability and climate change.	Identify opportunities to improve respiratory pathogen response mechanisms.	Identify key areas to update in national plan.	Test national pandemic influenza preparedness plan taking into account lessons learned from the COVID-19 pandemic and updated WHO global and regional guidance.
Pathogen scenario presented in simulation exercise	A cat influenza virus becomes transmissible to humans and causes a global pandemic. Cases are detected in nearby countries prior to the availability of vaccines. When the disease reaches the Cook Islands, it has a serious impact.	A novel influenza virus is detected in a neighboring country and spreads to Costa Rica. As hospitalizations and deaths increase, health facilities experience shortages of supplies and personal protective equipment. Fake news spreads widely, causing panic in the population.	A novel influenza outbreak occurs in a country on a different continent with human-to-human transmission documented. An increasing number of people are being hospitalized with severe respiratory disease.	An avian influenza outbreak occurs at a lake near Mongolia’s capital city of Ulaanbaatar. The outbreak quickly spreads to humans, with human-to-human transmission spreading across Mongolia, leading to a global pandemic.
Technical areas highlighted in simulation exercise	(1) Coordination, (2) communication, (3) surveillance, (4) public health and social measures, (5) clinical care, and (6) vaccines.	(1) Multi-sectoral and multi-level coordination, (2) risk communication and community engagement, and (3) triggers for operational decision-making.	(1) Multi-sectoral and multi-level coordination, (2) risk communication and community engagement, and (3) triggers for operational decision-making.	(1) Multi-sectoral and multi-level coordination, (2) risk communication and community engagement, and (3) triggers for operational decision-making.
Lessons learned	(1) The COVID-19 response provides valuable lessons that should be more systematically captured and translated into policies and practices that will improve the response to the next pandemic. (2) Decision-making about future preparedness activities should take into account budget requirements, methods for undertaking risk assessments, and strategies for ensuring sustainability. (3) Since there is a very limited national budget for health activities, it is imperative to identify the correct priorities in regard to pandemic preparedness.	(1) Roles of technical teams responsible for coordination and leadership of pandemic response are not clearly defined. (2) The draft plan has an overly narrow focus on the health sector and lacks mechanisms for multisectoral pandemic planning and response. (3) There is low awareness of the national risk communication plan. (4) The draft plan does not sufficiently elaborate procedures to increase capacities of health personnel. (5) The draft plan lacks a monitoring and evaluation component.	(1) Roles of multiple actors involved in pandemic response are not clearly defined. (2) Decision-making at national level remains opaque. (3) Sub-national and local level coordination is missing. (4) The Ministry of Public Health is not sufficiently engaged in the risk communication and community engagement committee that is currently being guided by other stakeholders. (5) Nongovernmental organizations are not optimally integrated into pandemic responses.	(1) Greater clarity is needed about which government agency leads in responding to an avian influenza outbreak that spreads to humans. (2) Greater clarity is needed about how the pandemic influenza preparedness plan fits within Mongolia’s overall disaster management framework. (3) Journalists need more education on misinformation and disinformation. (4) Resource management, funding, and political commitment are important considerations in pandemic preparedness planning.
Next steps proposed	(1) Update the national respiratory pathogen pandemic preparedness plan to capture lessons learned from the exercise and incorporate updated WHO guidance. (2) Circulate updated draft plan to stakeholders in preparation for seeking the approval of the cabinet of the national government. (3) Assess the opportunity costs associated with options for future pandemic preparedness activities and determine which of these activities would yield sufficient benefit to warrant funding. (4) Develop a separate national respiratory pathogen pandemic preparedness action plan with prioritized preparedness activities including budgets.	(1) Update the national respiratory pathogen pandemic preparedness plan incorporating recommendations from SimEx and PRET Module 1. (2) Link the plan to other key policy instruments and coordinating bodies in a fully integrated national disaster risk management system. (3) Clearly define mechanisms for coordinating intersectoral action. (4) Foster institutional and community-level awareness of the plan. (5) Reactivate the Inter-institutional Risk Communication Commission and review and disseminate the national health risk communication strategy.	(1) Synchronize the national pandemic plan with Lebanon’s National Health Strategy. (2) Improve multisectoral coordination, including through the clarification of roles and responsibilities. (3) Review and update the national health risk communication strategy. (4) Maintain a roster of reliable sources of information. (5) Consider how to strengthen coordination at subnational levels with entities such as municipalities, schools, universities, businesses and nongovernmental organizations.	(1) Develop a high-level multisectoral planning group to drive the strategic direction of national pandemic preparedness, and ensure continued engagement of all relevant agencies. (2) Develop a technical working group to manage updates of the pandemic preparedness plan based on recommendations from the simulation exercise and the workshop at which it was conducted. (3) Expand the scope of the pandemic preparedness plan from influenza to respiratory pathogens. (4) Develop a risk communication plan as an adjunct to the pandemic preparedness plan.
National plan(s) guiding response to simulation exercise	Draft Cook Islands Respiratory Pathogen Pandemic Preparedness Plan	Draft National Pandemic Plan for Influenza, SARS-CoV-2 and other Respiratory Viruses	National Pandemic Preparedness and Response Plan	Pandemic Influenza Preparedness and Response Guideline
World Health Organization region	Western Pacific Region	Region of the Americas	Eastern Mediterranean Region	Western Pacific Region
Population	15,040^1^	5,180,829^2^	5,489,739^2^	3,457,548^3^
Country income level	High-income^4^	Upper-middle income^5^	Lower-middle income^5^	Lower-middle income^5^
Date of simulation exercise	27–28 March 2023	26–27 July 2022	18 August 2022	19–20 April 2023
Number of exercise participants	57	21	30	37

1 Cook Islands Statistics Office (https://stats.gov.ck/census-2021-updates/).

2 World Bank data (https://data.worldbank.org/).

3 Mongolian Statistical Information Service (https://www.1212.mn/en).

4 Ministry of Finance and Development, Government of the Cook Islands (https://www.mfem.gov.ck/international-development-assistance).

5 World Bank country and lending groups, fiscal year 2024 (https://datahelpdesk.worldbank.org/knowledgebase/articles/906519-world-bank-country-and-lending-groups).

WHO, World Health Organization.

## 3 Outcomes

### 3.1 Overview of exercise implementation

Exercise PanPRET-1 was implemented in Costa Rica, Cook Islands, Lebanon and Mongolia between July 2022 and April 2023 (Table 1). The exercises in Costa Rica, Lebanon and Mongolia were designed to explore the technical areas that were the focus of the base scenario. The Cook Islands exercise was designed to address those areas as well as surveillance; public health and social measures; clinical care; and vaccines. Across the four countries, the number of exercise participants ranged from 21 to 57. Government sectors and technical areas represented in the exercises included human health, animal health, disaster management, emergency coordination, risk communication, hospital management, education, defense and border control. Cook Islands also had nongovernmental sector participation from volunteer service organizations.

### 3.2 Country findings

In the Cook Islands, planners structured the exercise to encourage participants to draw on the country’s COVID-19 experiences while assessing the functionality of a newly drafted respiratory pathogen pandemic plan. The exercise scenario described an overseas cat influenza outbreak that resulted in a global pandemic among humans. During the COVID-19 pandemic, there were no reported cases in the Cook Islands until February 2022, well after vaccines were available to the population. In the exercise scenario, the hypothetical virus reached Cook Islands before a vaccine had been developed, providing participants with the opportunity to explore a very different pandemic situation. Participants found it difficult to determine whether authorities in the animal or human health sector would coordinate the early response stages. Participants observed that many experiences relating to COVID-19 could inform responses to future pandemics, even if pandemic characteristics were different. For example, COVID-19 learnings pointed to the need for risk communication strategies that take into account unreliable internet access and different information needs across culturally diverse communities. Participants concluded that learnings derived from both successes and failings in the national response to COVID-19 should be systematically captured and translated into policy and operational improvements. They also called attention to the challenge of determining which pandemic preparedness activities should be prioritized in the context of resource limitations.

In Costa Rica, the exercise scenario was based on the detection of a novel influenza virus in a neighboring country. Exercise participants explored how effectively the Draft National Pandemic Plan for Influenza, SARS-CoV-2 and other Respiratory Viruses guided their response to this scenario. Regarding multisectoral coordination and communication, a lack of cohesion in the overall health policy framework was identified. It was noted that the respiratory pandemic plan should be linked to Costa Rica’s National Action Plan for Health Security and Strategy for Health Risks, Disasters and Emergencies. Participants further identified a need for mechanisms to coordinate respiratory pathogen pandemic preparedness and response across all relevant sectors including the private sector. Regarding RCCE, many workshop participants were not familiar with the national Health Risk Communication Strategy. Participants called for this strategy to be reviewed and widely disseminated, and also for Costa Rica’s Inter-institutional Risk Communication Commission to be reactivated. Regarding triggers for escalating and de-escalating activities, participants recommended developing operational flowcharts that include clear definitions of the functions of implementing entities. They also called for greater clarity about the roles of the technical teams engaged in the response.

In Lebanon, the exercise was designed to inform ongoing efforts to strengthen intersectoral preparedness and response efforts under the One Health approach. Implementation of the exercise was viewed as a key step in developing a more contextualized national pandemic preparedness plan. In the course of considering the scenario and injects, participants frequently made reference to lessons learned across the different stages of Lebanon’s COVID-19 response. It was observed that while the Ministry of Public Health leads risk assessments as well as preliminary preparedness and response efforts in the early stages of a pandemic, the Supreme Defense Council plays a progressively larger decision-making role with the escalation of the situation. Participants noted that heterogeneous areas of Lebanon’s health sector need to be well-mapped at the national and subnational levels in order to optimize the use of available resources. Participants also discussed the importance of fostering institutional trust and compliance with recommended public health and social measures through effective risk communication. It was suggested by participants that efforts in this regard should be channeled toward the objectives of improving community engagement and identifying reliable media and information sources.

The exercise in Mongolia was designed to test the existing national pandemic influenza preparedness plan while also taking into account lessons learned from the COVID-19 pandemic and revised WHO global and regional guidance. The exercise scenario described an avian influenza outbreak that began in Mongolia and spread widely through human-to-human transmission, resulting in a global pandemic. Challenges that participants identified included a lack of clarity about which government agency should lead the response to an outbreak that began in animals and spread to humans, as well as uncertainty about how the existing national plan fit within Mongolia’s overall disaster management framework. Participants highlighted the need to estimate financial, human and other resource requirements and improve resource management in future pandemics. They also noted the importance of effective risk communication and observed that it may be beneficial to develop a separate risk communication plan. Participants considered the merits of using a pathogen-specific approach versus a mode-of-transmission approach to pandemic planning. As of this writing, stakeholders in Mongolia were working to determine how the scope of the pandemic plan would be revised accordingly. Participants recommended establishing a high-level multisectoral planning group to guide national pandemic preparedness efforts, with the Deputy Prime Minister serving as the chair of this group.

### 3.3 Exercise evaluation

**Figure 1:**
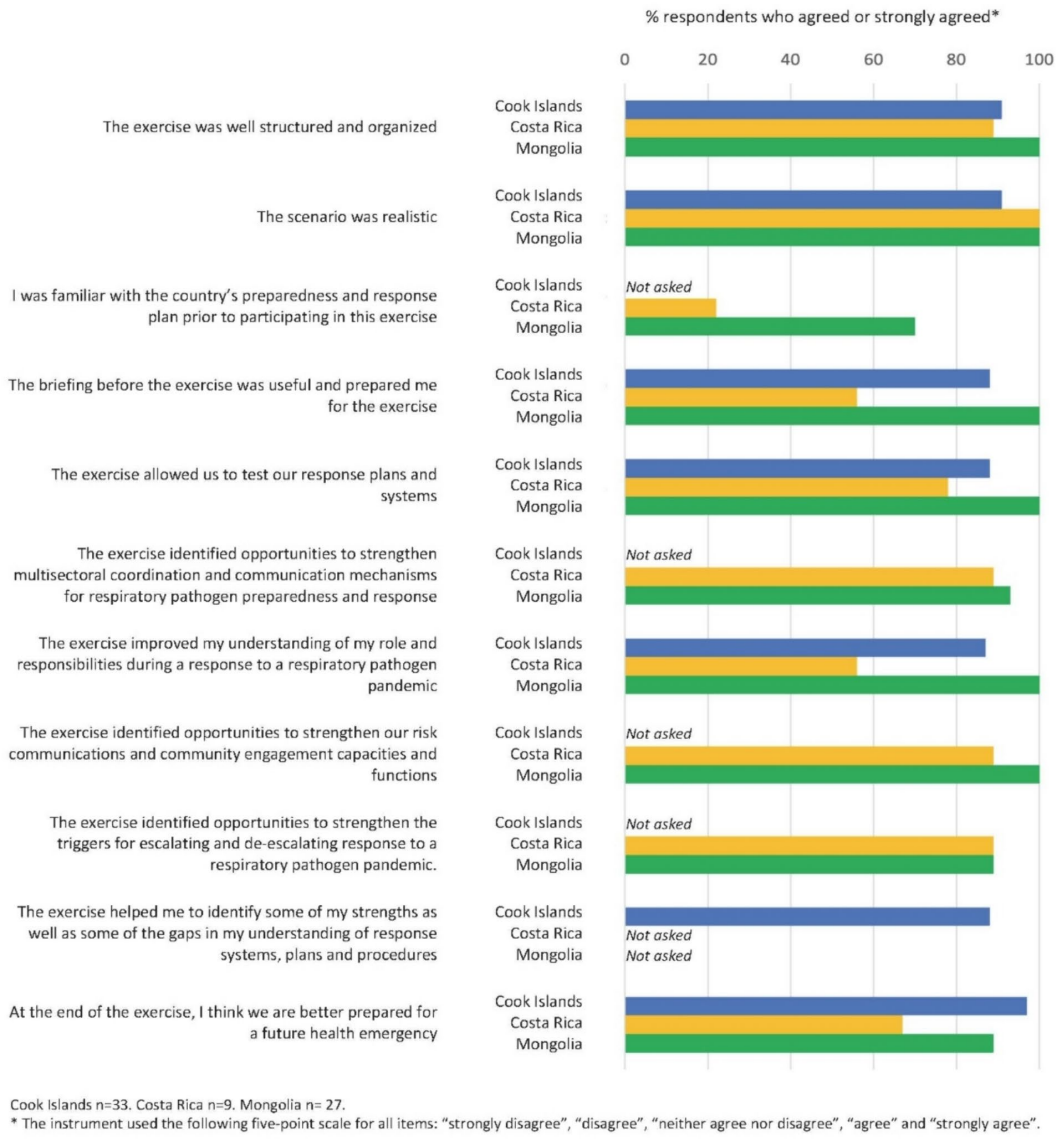
PanPRET-1 exercise evaluations in Cook Islands, Costa Rica, and Mongolia.

Exercise participants in Cook Islands, Costa Rica and Mongolia completed quantitative evaluations (Figure 1) after their participation. High proportions of participants in all three countries agreed or strongly agreed that multiple aspects of the exercise were well designed and were beneficial. In Costa Rica and Mongolia, more than 85 percent of participants responded positively in regard to the value of the exercise for strengthening multisectoral coordination and RCCE capacities. In Cook Islands and Mongolia, more than 85 percent of participants agreed or strongly agreed that “at the end of the exercise, I think we are better prepared for a future health emergency.” Qualitative feedback from participants in Lebanon also indicated that the exercise was perceived to make meaningful contributions to respiratory pathogen pandemic preparedness and response. In particular, one participant noted that “this exercise could be replicated at the local level to reinforce Lebanon’s coordination mechanisms between national and local levels.” Participants in Lebanon also highlighted multiple critical actions for strengthening preparedness including developing more tangible pandemic plans and conducting periodic simulation exercises based on Exercise PanPRET-1 that bring together multiple levels and sectors.

## 4 Discussion

To support countries in reviewing and developing national pandemic plans using a mode-of-transmission approach, WHO has introduced the first PRET module on respiratory pathogen pandemic planning along with supporting tools such as Exercise PanPRET-1 (1). The experiences of Exercise PanPRET-1 national implementers in Cook Islands, Costa Rica, Lebanon and Mongolia have demonstrated that this tool can be effective for testing pandemic plans in a multisectoral forum and identifying ways to improve key components of preparedness and response.

Across the four countries, the exercise drew attention to the value of mode-of-transmission planning. In particular, countries saw opportunities to build on pathogen-specific planning and strengthen common systems that could be utilized for influenza, coronaviruses, and other respiratory pathogens. In the debriefing that followed the Mongolia exercise, participants emphasized the potential for a mode-of-transmission approach to promote more efficient capacity-building across Mongolia’s multisectoral pandemic preparedness system and recommended convening a multisectoral planning group to lead the country in this direction. Similarly, the exercise in Lebanon highlighted the utility of a mode-of-transmission approach for specific components of preparedness and response systems such as RCCE. It was noted that holistically building community trust on respiratory pathogen public health and social measures would be more effective than taking disease-specific approaches. Exercise participants in all four countries identified high-level political commitment as a critical pathway forward. They stressed the value of including diverse sectors in pandemic preparedness and response activities, including academia, philanthropy, civil society and the private business sector.

Country exercises also highlighted opportunities for mode-of-transmission planning to build on learnings from COVID-19. Participants in Costa Rica recommended making improvements to decision-making algorithms that were used to guide the country’s COVID-19 response and are also applicable to other respiratory pathogen outbreaks and epidemics. Additionally, countries discussed ways of institutionalizing emerging technical areas that were established during the COVID-19 pandemic. This includes infodemic management in Lebanon and Mongolia, where participants identified the need to proactively manage mis- and disinformation by engaging more systematically with community and media groups to strengthen capacities for health journalism.

The exercise brought to light notable challenges associated with the high-level coordination of multifaceted responses to public health emergencies, consistent with findings from other simulation exercises and with lessons learned from the COVID-19 pandemic (3, 7, 12, 13). In particular, participating countries found that centralized leadership and decision-making was critical for coordinating national responses. Following the COVID-19 pandemic, it was recognized that conducting simulation exercises which included sectors beyond health would strengthen coordination mechanisms and enable countries to be better prepared to deliver large-scale response operations (12, 13). In addition, countries noted that public messaging on pandemic risks and community engagement should be targeted in order to build trust, facilitate tailored community-led responses, and address mis- and dis-information (12, 13).

Exercise participants in some countries commented on the need for pandemic plans to align more fully with other elements of their countries’ health emergency management policy architecture. Participants in two countries reported that greater clarity is required in regard to the respective roles of the animal and human health sectors when a pathogen of concern is spreading among both animals and humans. More generally, uncertainty about how to coordinate the work of various entities involved in responding to pathogen-driven public health threats was observed in all four countries. The complex nature of infodemic management also presented challenges, with exercise participants grappling with how best to adapt traditional risk communication approaches to internet and social media platforms that target diverse communities and age groups.

Findings overall suggest that Exercise PanPRET-1 is a versatile tool for engaging stakeholders in determining how to strengthen pandemic planning in a wide range of national settings. Exercise PanPRET-1 thus can help facilitate global implementation of the International Health Regulations (2005) Monitoring and Evaluation Framework. This framework includes the mandatory States Parties self-assessment annual reporting as well as three optional elements: voluntary external evaluation, after-action reviews, and simulation exercises (14).

As such, countries are encouraged to:

- Implement Exercise PanPRET-1 in a manner that fully integrates the activity into the ongoing planning cycle (1). This can be achieved by bringing together multiple sectors under health sector leadership through the implementation of Exercise PanPRET-1 at all levels, and updating pandemic preparedness plans using a mode-of-transmission approach and exercise recommendations.
- Use Exercise PanPRET-1 as a catalyst to strengthen common systems for pandemic preparedness and response, and to advocate for high-level ongoing national political commitment in the pandemic planning cycle.
- Use Exercise PanPRET-1 as the basis to systematically and periodically test multi-sectoral approaches to pandemic preparedness capacities across additional technical areas and scenarios not currently covered by the package. This can be achieved by adapting the scenario, based on national requirements, to focus on other technical areas including but not limited to public health and social measures, vaccine deployment, clinical management and/or partner engagement.

Quantitative and qualitative feedback suggested that the value of the exercise may be enhanced by ensuring that all participants are pre-briefed about relevant policies and plans in their respective countries. Potential bottlenecks to following through on exercise recommendations, such as a codifying how accountability for proposed activities will be shared among multiple agencies, should be identified and addressed in post-exercise action plans.

Limitations to these Exercise PanPRET-1 findings include the small country sample and the low level of participation from outside of the government sector. Additionally, the selection of exercise participants may have been biased by factors such as the nature of existing multisectoral collaborations addressing pandemic preparedness in the four countries, and the availability of appropriate technical focal points from participating Ministries. Quantitative participant feedback was only available from three of the four countries, and in the future can be improved by including questions on sector affiliation and roles.

In conclusion, Exercise PanPRET-1 supports countries in addressing pandemic preparedness and response planning through a mode-of-transmission approach and identifying next steps for making improvements. WHO is expanding the applications of Exercise PanPRET-1 by incorporating additional technical areas into the exercise and developing versions for specific stakeholder groups such as for civil society and international partner organizations. The engagement and effective coordination of different types of multisectoral actors and technical teams, including under a One Health framework, is key to strengthening mode-of-transmission pandemic planning. High-level political commitment is also required. Conducting TTX and monitoring the implementation of action plans based on exercise findings is a valuable mechanism for helping stakeholders develop and implement a shared country-owned vision for pandemic preparedness and response. Countries are encouraged to incorporate TTX such as Exercise Pan-PRET-1 into a continuous cycle of activity to improve respiratory pandemic preparedness and response planning.

## Data availability statement

The original contributions presented in the study are included in the article/supplementary material, further inquiries can be directed to the corresponding author.

## Author contributions

HC: Conceptualization, Methodology, Project administration, Supervision, Validation, Writing – review & editing. OA: Conceptualization, Methodology, Project administration, Validation, Writing – review & editing. RA: Writing – review & editing. MA: Writing – review & editing. MB: Writing – review & editing. AB: Writing – review & editing. HB: Writing – review & editing. DB: Writing – review & editing. UC: Writing – review & editing. GD: Writing – review & editing. OD: Writing – review & editing. IG: Writing – review & editing. NG: Writing – review & editing. RH: Writing – review & editing. AJ: Writing – review & editing. SN: Writing – review & editing. KN: Writing – review & editing. PN: Writing – review & editing. BP: Writing – review & editing. N-ET: Writing – review & editing. AV: Writing – review & editing. VW: Writing – review & editing. KS-H: Conceptualization, Methodology, Validation, Writing – original draft, Writing – review & editing. SG: Writing – review & editing. SB: Conceptualization, Supervision, Writing – review & editing. GS: Conceptualization, Methodology, Project Administration, Supervision, Writing – review & editing.

## References

[1] World Health Organization. Preparedness and resilience for emerging threats module 1: planning for respiratory pathogen pandemics. (2023). Available at: https://www.who.int/publications/i/item/9789240084674

[2] World Health Organization. Strengthening pandemic preparedness planning for respiratory pathogens: policy brief, 27 April 2022. (2022). Available at: https://www.who.int/publications/i/item/WHO-2019-nCoV-Policy_brief-pandemic_preparedness-2022.1

[3] Copper FA Mayigane LN Pei Y Charles D Nguyen TN Vente C. Simulation exercises and after action reviews – analysis of outputs during 2016-2019 to strengthen global health emergency preparedness and response. Glob Health. (2020) 16:115. doi: 10.1186/s12992-020-00632-w, PMID: 33261622 PMC7705853

[4] European Centre for Disease Prevention and Control. Handbook on simulation exercises in EU public health settings: how to develop simulation exercises within the framework of public health response to communicable diseases. (2014). Available at: https://www.ecdc.europa.eu/en/publications-data/handbook-simulation-exercises-eu-public-health-settings

[5] World Health Organization. WHO simulation exercise manual: a practical guide and tool for planning, conducting and evaluating simulation exercises for outbreaks and public health emergency preparedness and response. (2017). Available at: https://apps.who.int/iris/handle/10665/254741

[6] Center for Health Security, Johns Hopkins Bloomberg School of Public Health. Event 201. (2023). Available at: https://centerforhealthsecurity.org/our-work/tabletop-exercises/event-201-pandemic-tabletop-exercise#scenario

[7] Alves F Artursson K Bloch J Brisabois A Imberechts H Jokelainen P. A multi-country one health foodborne outbreak simulation exercise: cross-sectoral cooperation, data sharing and communication. Front Public Health. (2023) 11:1121522. doi: 10.3389/fpubh.2023.1121522, PMID: 37383258 PMC10293640

[8] World Health Organization. South Sudan conducts an Ebola tabletop exercise. (2019). Available at: https://www.afro.who.int/news/south-sudan-conducts-ebola-tabletop-exercise

[9] World Health Organization. Préparation et riposte aux épidémies: Des exercices de simulations pour évaluer le dispositif contre une éventuelle flambée de choléra. (2018). Available at: https://www.afro.who.int/node/10511

[10] Dausey DJ Buehler JW Lurie N. Designing and conducting tabletop exercises to assess public health preparedness for manmade and naturally occurring biological threats. BMC Public Health. (2007) 7:92. doi: 10.1186/1471-2458-7-92 17535426 PMC1894789

[11] World Health Organization. Pandemic influenza preparedness in WHO member states: report of a member states survey. (2019). Available at: https://apps.who.int/iris/handle/10665/325411

[12] European Centre for Disease Prevention and Control. Lessons from the COVID-19 pandemic. (2023). Available at: https://www.ecdc.europa.eu/sites/default/files/documents/COVID-19-lessons-learned-may-2023.pdf

[13] World Health Organization. Learnings from COVID-19 for future respiratory pathogen pandemic preparedness: a summary of the literature. (2024). Available at: https://www.who.int/publications/i/item/9789240086531

[14] World Health Organization. International health regulations (2005) monitoring and evaluation framework. (2024). Available at: https://www.who.int/publications/i/item/international-health-regulations-(-2005)-ihr-monitoring-and-evaluation-framework

